# Co-Evolution of the Mating Position and Male Genitalia in Insects: A Case Study of a Hangingfly

**DOI:** 10.1371/journal.pone.0080651

**Published:** 2013-12-03

**Authors:** Qionghua Gao, Baozhen Hua

**Affiliations:** State Key Laboratory of Crop Stress Biology for Arid Areas, Key Laboratory of Plant Protection Resources and Pest Management of the Education Ministry, Entomological Museum, Northwest A&F University, Yangling, Shaanxi, China; University of Melbourne, Australia

## Abstract

Hangingflies are unique for the male providing a nuptial gift to the female during mating and taking a face-to-face hanging copulation with the female. Their male genitalia are peculiar for an extremely elongated penisfilum, a pair of well-developed epandrial lobes (9th tergum), and a pair of degenerated gonostyli. However, the co-evolution of their face-to-face copulation behavior and the male genitalia has rarely been studied hitherto. In this paper the mating behavior of the hangingfly *Bittacus planus* Cheng, 1949 was observed under laboratory conditions, and the morphology of the male and female external genitalia was investigated using light and scanning electron microscopy. The male provides an insect prey as a nuptial gift to the female in courtship and mating process, and commits a face-to-face copulation. During copulation, the male abdomen twists temporarily about 180° to accommodate their face-to-face mating position. The aedeagal complex has an extremely elongated penisfilum, corresponding to the elongated spermathecal duct of the female. The well-developed epandrial lobes serve as claspers to grasp the female subgenital plate during copulation, replacing the function of gonostyli, which are greatly reduced in Bittacidae. The modified proctiger assists the penisfilum to stretch and to enter into the female spermathecal duct. The possible reasons why this species might mate face-to-face are briefly discussed.

## Introduction

In most animal species with internal fertilization, male genitalia are among the most diverse, complex, and rapidly evolving morphological structures [1]–[3]. Because of the extraordinary diversity in form and function, genital structures are often used to determine species status of insects and to estimate the evolutionary relationships among insects, including hangingflies [4]–[8]. However, how the genitalia of insects arrived at their current configuration and the driving force of genital evolution are poorly understood.

Previous studies highlight three main hypotheses to explain the evolutionary processes responsible for genital evolution: the lock-and-key hypothesis, the pleiotropy hypothesis and the sexual selection hypothesis [9]. The lock-and-key hypothesis proposes that female genital structures are complex mechanical locks, into which only males of the same species can fit [10]. Although this hypothesis sounds elegant to explain why genitalia are often species-specific in form, the supposed female locks rarely exist [10]–[13]. The pleiotropy hypothesis states that genital evolution is an indirect result of evolution of genetically correlated characters through accumulated pleiotropic effects of genes that code for both genital and general morphology [14]. Again, this hypothesis is not currently well supported [9], [15]. The more convincing alternative hypothesis is that the evolution of species-specific genitalia arises through sexual selection and/or sexual conflict. This hypothesis proposes that males and females are engaged in co-evolutionary arms races over control of copulation and insemination and includes three main mechanisms [9]: sperm competition, cryptic female choice, and sexual conflict. The sperm competition assumes that male genitalia may be reflected in differences in ability to displace or dislocate sperm from previous males within the female reproductive tract or to induce nonreceptivity in females [9], [16]. The cryptic female choice states that male genitalia may differ in their ability to stimulate multiply-mated females to selectively use sperm from males with superior stimulatory capabilities of genital morphology over that of others to fertilize their eggs [1], [17]. Sexual conflict occurs when the evolutionary interests of the two sexes are at odds [18]–[23], and may generate co-evolution between the two sexes in the rapid specialization and modification of certain traits, particularly structures involved in mating [21]. Many behavioral adaptations are involved in mediating sexual selection and sexual conflict. The provision of a nuptial gift prior to and during mating is an interesting strategy employed by male hangingflies (Mecoptera: Bittacidae) in their pursuit of a mate [24], [25].

The Bittacidae are commonly called hangingflies because the adults are unable to stand on a surface but hang on the edges of leaves or twigs of plants by their prehensile forelegs [26]. During courtship and copulation, the male usually provides an insect prey as a nuptial gift to the female [27], and mate with the female in a face-to-face hanging position, with their forelegs suspended from a twig or leaf [28]. The sexual behavior of Bittacidae has been well studied in the North American *Hylobittacus apicalis*, *Bittacus strigosus*, *B. pilicornis* and *B. stigmaterus* [15], [24], [29]–[35], the Australian *Harpobittacus australis*, *Hp. nigriceps*, and *Hp. similis* [27], [36]–[40], the European *B. italicus* [28], the Japanese *B. mastrillii* [41], [42], and the Chinese *B. planus* [43]. However, these studies are generally concentrated on behavioral observations only (except Mickoleit and Mickoleit (1978), who describe both courtship and copulation behavior in *B. italicus* and explore variation in genital morphology [28]).

The male genitalia in Bittacidae are unique in Mecoptera by an extremely elongated penisfilum, a pair of well-developed epandrial lobes (9th tergum), and a pair of greatly degenerated gonostyli [44], [45]. This raises two currently unanswered questions: why are their genitalia structured like this and is there a relationship between their face-to-face copulation and these peculiar male genitalia?

In this study we explore the co-evolutionary knowledge of the face-to-face copulation and the functional morphology of male genitalia in the hangingfly *Bittacus planus* Cheng, 1949 through observations of courtship and copulation behavior under laboratory conditions and via morphological observations using light and scanning electron microscopy.

## Materials and Methods

### Ethics Statement

No specific permits were required for the described field studies: a) no specific permissions were required for these locations/activities; b) locations were not privately-owned or protected; and c) the field studies did not involve endangered or protected species.

### Insect Collection

*Bittacus planus* Cheng, 1949 is the dominant hangingfly species in the Qinling Mountains, Shaanxi Province of central China. Adults of *B. planus* were collected from the Huoditang Forest Farm, Zhuque Forest Park, and the Jialing River Source in the Qinling Mountains in late July to early August from 2009 to 2012, and the Baihua Forest Farm in Gansu Province in early August 2011.

### Insect Rearing

The adults were all placed in a nylon gauze cage (40×40×60 cm) for mating behavior observation. Live flies (blow flies, house flies and fruit flies), crane flies, moths and other flying insects captured from fields were supplied as food items. Each hangingfly was supplied three to four prey items per day. Twigs of plants were placed in the cage to simulate the shaded micro-environment for the hangingflies. The cage was cleaned regularly (usually at 8:00 am and 20:00 pm) every day; old twigs were substituted with fresh twigs; a fine spray of water was provided to maintain the humidity in the cage; and newly captured food items (see above) were supplied.

### Mating Behavior Observation

A total of 15 females were observed as they interacted with nine males possessing different sizes of nuptial prey (Mean  = 21.90±1.72 mm^2^, *n* = 70) during 16–31 July 2012. Each phase of the mating behavior and its duration were recorded. Photographs were taken with a Canon EOS 550D digital camera.

The surface area of the nuptial prey was measured as follows: the length from the front of the head to the distal end of the abdomen and the widest or highest (depending upon which was larger) portion of the thorax or abdomen [34].

### Light and Scanning Electron Microscopy

Fifty adults (30 males and 20 females) were used for morphological studies.

For light microscopy, live adults were fixed in Dietrich's fluid (formalin: 95% ethanol: glacial acetic acid: distilled water  = 6∶15∶1∶80, v/v) before being preserved in 75% ethanol. The genitalia were dissected under a Nikon SMZ1500 Stereoscopic Zoom Microscope. Photographs were taken with a CCD digital camera (Retiga 2000R, Q-Imaging) equipped on the microscope and further treated with the software Auto-Montage Pro.

For scanning electron microscopy (SEM), live adults were fixed in a mixture of 2% paraformaldehyde and 2.5% glutaraldehyde in phosphate buffer (PB, 0.1 mol/L, pH 7.2) for 12 h at 4°C. Before dehydration in a graded ethanol series, they were ultrasonically cleaned for 30 s. Then the materials were dried in a critical-point drier with liquid CO_2_, coated with gold in a sputter coater, and examined in a Hitachi S-3400N scanning electron microscope (Hitachi, Tokyo, Japan) at 15 kV.

## Results

### Courtship and Copulation Behavior

Provided that the food supply is sufficient, the courtship and copulation of *B. planus* occurred mainly from the dusk to the dawn under laboratory conditions. The sequence of courtship and copulation behaviors began when the male caught a prey item (or stole one from another male). The mating process was divided into four phases: searching, calling, attaching, and copulating phases.

**Figure 1:**
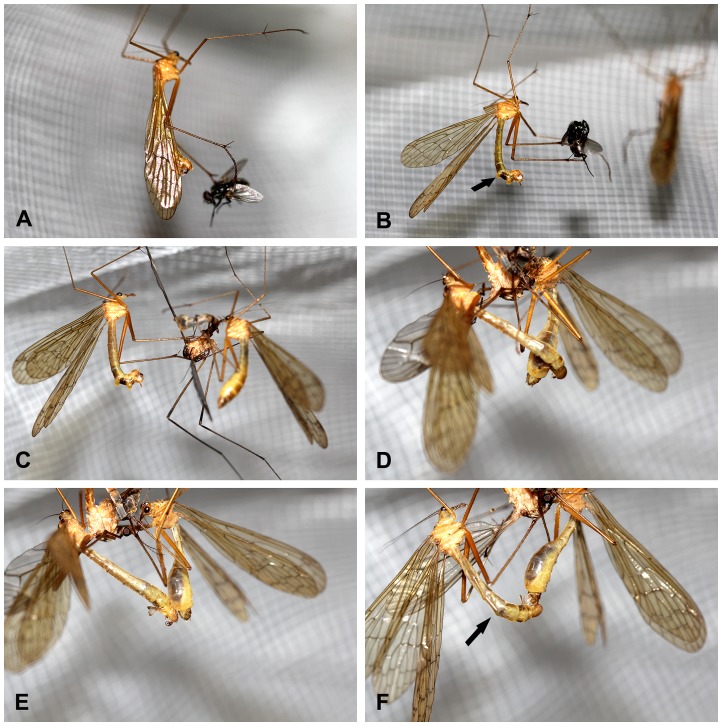
The copulatory process of *Bittacus planus*. (A) Searching phase, the male makes a short flight to search females in vicinity. (B) Calling phase, the male everts his paired bifurcated sex pheromone glands between segments VI and VII and segments VII and VIII in a slow rhythmic motion to release species-specific sex pheromone to attract females nearby, arrow shows the male sex pheromone glands. (C)–(F) Attaching phase (the left: male; the right: female): (C) the male presents the prey as a nuptial gift to the female with his sex pheromone glands still everted and begins the attaching phase; (D) the female accepts the nuptial prey, and begins to assess its size and palatability while the male attempts to reach out his abdomen to locate her genitalia; (E) the female shows her mating desire by bending her abdomen backward and the male makes his epandrial lobes widely opened and his genitalia inflated; (F) the male temporarily twists his abdomen along his longitudinal axis up to 180° to adapt to the face-to-face mating with female in hanging position, arrow shows the 180° twisting of the male abdomen. Pictures in (A, B) and (C–F) were taken from different couples.

Before mating, the male captured a flying insect with his middle and/or hind legs, inserted his mandibulate mouthparts into the prey body likely to inject some salivary secretions to paralyze the prey, and fed upon the prey for a short while to taste its palatability. The surface area of 16 mm^2^ or more for the prey items were generally accepted by the females, e.g. blow flies and crane flies [35]. If the prey was too small (<16 mm^2^, e.g. fruit flies) or unpalatable (e.g. lady beetles), the male usually discarded the prey and obtained a new one. If the prey was large enough and palatable, he retained it and initiated searching behavior. During this searching phase, the male made a short flight to search for any females in the vicinity and sought suitable twigs or leaves of plants to hang on (Fig. 1A).

Once hanging, the male bent his distal abdomen ventrally into a J-shape commencing from the sixth segment, entering into the calling phase (Fig. 1B). A calling male everted a pair of bifurcated sex pheromone glands (situated between abdominal segments VI and VII and segments VII and VIII) in a slow rhythmic motion to release sex pheromones that attracted females nearby (Fig. 1B). During this period, the male genitalia were fully inflated with their epandrial lobes widely open to attract females visually as well as chemically. When one or more females in vicinity approached, a calling male terminated pheromone emission by retracting his glands. Occasionally he retracted his pheromone glands once a female had accepted the nuptial prey item. If a female accepted the prey item, the two individuals approached each other and suspended themselves on vegetation by their prehensile front tarsi. The male presented the prey as a nuptial gift to the female soon (Fig. 1C), and entered into the attaching phase.

**Figure 2:**
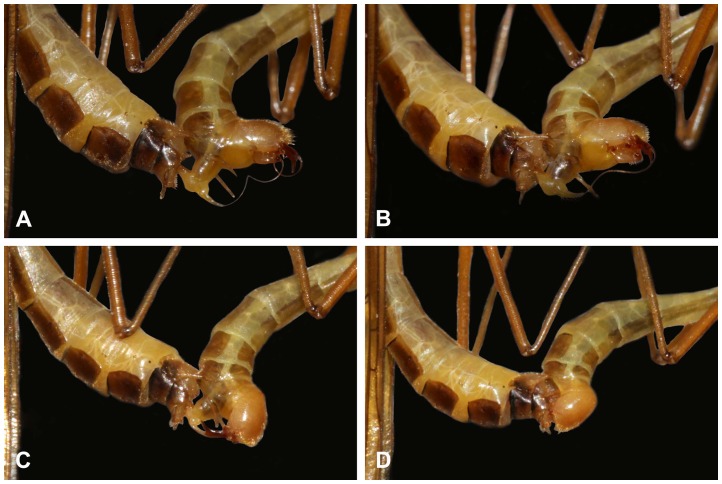
The copulating phase of *B. planus*, showing the gradually insertion process of the male penisfilum (the left: female; the right: male). (A) The male firmly holds the subgenital plate of the female by his epandrial lobes and the elongated penisfilum is stretched by the upper and lower branches of the proctiger. (B) The proctiger assists the entering of the male penisfilum into the spermathecal duct of the female. (C) The partly inserted penisfilum. (D) The male penisfilum is fully inserted.

**Figure 3:**
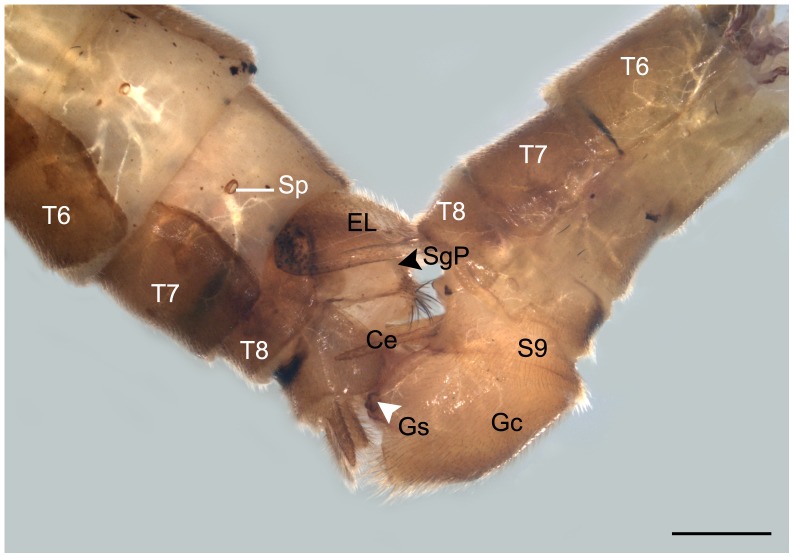
Light micrographs of a copulation pair of *B. planus*, lateral view. The white arrowhead shows the gonostylus of the male, the black arrowhead shows the female subgenital plate. Ce, cercus; EL, epandrial lobe; Gc, gonocoxite; Gs, gonostylus; S9, sternum IX; SgP, subgenital plate; Sp, spiracle; T6–8, terga VI to VIII. Scale bar  = 0.5 mm.

During the attaching phase, the female assessed the size and palatability of the nuptial gift. Simultaneously, the male attempted to reach out his abdomen with his epandrial lobes wide-open to seek the tip of the female abdomen and locate her genitalia (Fig. 1D). Under laboratory conditions, most females were very reluctant to mate with males carrying fruit flies (too small) and lady beetles (unpalatable). The female, after tasting the small or unpalatable nuptial offering, usually refused to mate with the male and took the gift away; the male attempted to retain the nuptial gift and if successful used it to secure a subsequent mating. By contrast, if the nuptial gift was accepted, the receptive female bent her abdomen (Fig. 1E). The male temporarily twisted his abdomen along the longitudinal axis up to 180° to allow him to adopt a face-to-face mating position with the female (Fig. 1F). A male can twist his abdomen either clockwise or anticlockwise. The male epandrial lobes served as claspers to slip backward and grasp the subgenital plate of the female (Fig. 1F). More precisely, black spines on the male epandrial lobes grasped the pleural region of the female 8th abdominal segment (Figs. 2 and 3). The copulating phase started when the male had a firm hold of the female subgenital plate.

During the copulating phase, the male genitalia were fully inflated. The extremely elongated spring-like penisfilum was stretched by the upper and lower branches of the proctiger. The male then attempted to insert his penisfilum into the opening of the spermathecal duct of the female with the aid of the upper branch of proctiger (Fig. 2A−C). With combined movements of the proctiger and aedeagal complex, the outstretched penisfilum was gradually inserted into the spermathecal duct of the female till the penisfilum was fully inside the reproductive tract of the female (Fig. 2D). When successfully coupled, the male genitalia fit those of the female closely (Fig. 3). Occasionally, the male and female formed an L-shape during copulation, which meant that the female was suspended by the male only through male genitalia.

After copulation, the male disengaged his genitalia from the female and immediately turned his abdomen back to the normal position. During this time, the male also struggled to obtain any uneaten prey remaining with the female. Usually the male retrieved it successfully, and fed on it (possibly to evaluate its size and nutritional value) and then he usually used it as a nuptial gift for further copulation attempts. Occasionally he discarded it and caught or stole a new prey item. If food resources were limited, a male could also retrieve a discarded prey item from the ground, and reused it as a nuptial gift to lure another female.

The copulation duration of *B. planus* ranged from a few minutes to several hours, depending on the size and nutritional value of the prey (data not provided). In general, the larger the nuptial gift, the longer the mating duration. The large nuptial gift can ensure the male to have successive mating attempts.

### Morphology of the Male Genitalia

The male genitalia of *B. planus* consist of a pair of dorsal epandrial lobes, a proctiger, a pair of ventral gonopods, and a central aedeagal complex.

#### The Epandrial Lobes and Proctiger

**Figure 4:**
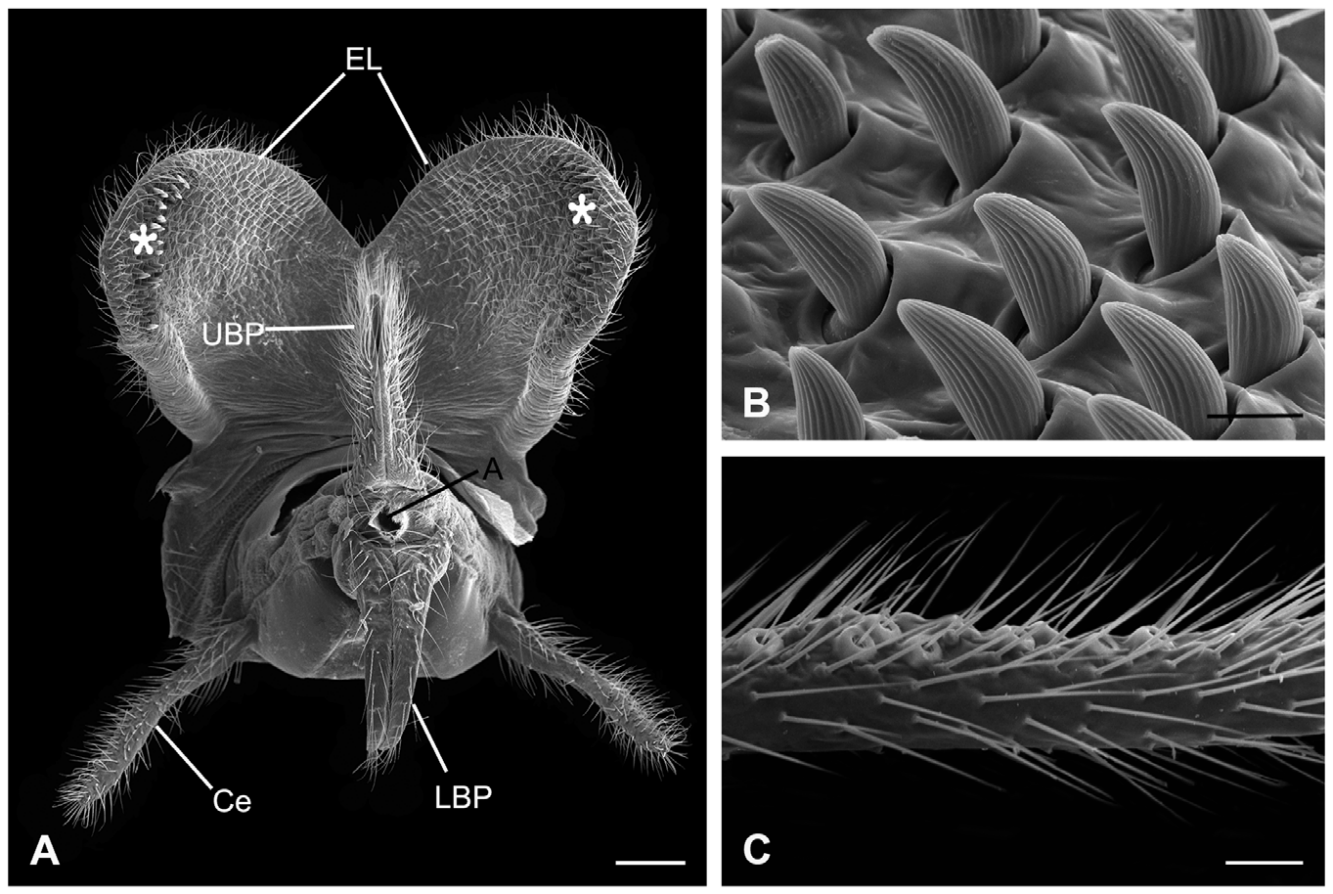
SEM micrographs of the male epandrial lobes, proctiger and cercus in *B. planus*. (A) Caudal view of the epandrial lobes and proctiger, showing the modified paired epandrial lobes and upper and lower branches of proctiger, the asterisks show stout spines which were regularly distributed along its inner posterior edge. (B) Magnification of the Inner view of the posterior margin of the epandrial lobes, showing the stout spines. (C) Magnification of cercus. A, anus; Ce, cercus; EL, epandrial lobe; LBP, lower branch of proctiger; UBP, upper branch of proctiger. Scale bars: (A)  = 200 μm; (B)  = 20 μm; (C)  = 50 μm.

The epandrial lobes are a pair of backward projecting structures modified from the ninth tergum, roughly quadrangular in lateral view (Figs. 3 and 4A). The two epandrial lobes coalesce at base and are furnished with stout spines regularly distributed along the inner posterior edge (Fig. 4A, B).

The proctiger consists of an upper branch and a lower branch (Fig. 4A). The digitate upper branch protrudes from between the epandrial lobes, with dense setae, especially on the distal part. The lower branch is broader at base and slender toward apex. The anus is situated centrally between the upper and lower branches. The unsegmented cerci are slender and somewhat clavate with long setae and various sensilla (Fig. 4A, C).

#### The Gonopods

**Figure 5:**
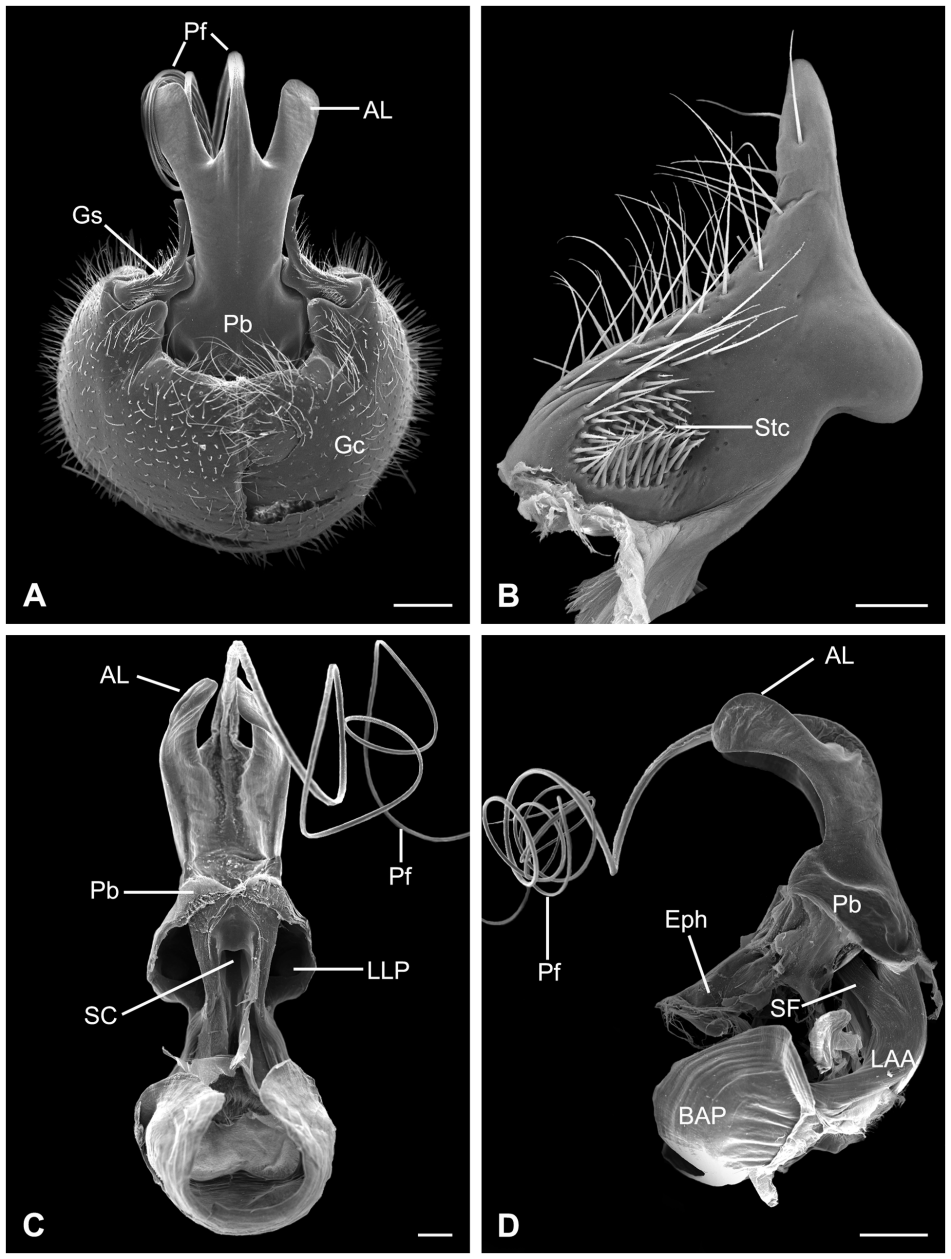
SEM micrographs of the male genitalia in *B. planus*. (A) Caudo-ventral view. (B) Ventral view of the left gonostylus, showing the stylocavernula, a bundle of sensilla chaetica. (C) Dorsal view of the aedeagal complex, with the epiphallus and the support of ejaculatory sac removed, showing the sperm channel. (D) Lateral view of aedeagal complex. AL, aedeagal lobe; BAP, basal apodeme of phallobase; Eph, epiphallus; Gc, gonocoxite; Gs, gonostylus; LAA, lateral arm of apodeme of phallobase; LLP, lateral lumen of phallobase; Pb, phallobase; Pf, penisfilum; SC, sperm channel (median lumen of phallobase); SF, supporting frame of sperm channel; Stc, stylocavernula. Scale bars: (A), (C) and (D)  = 200 μm; (B)  = 50 μm.

The two-segmented gonopods are paired appendages of the ninth abdominal segment. Each gonopod consists of a well-developed basal gonocoxite and a much-reduced distal gonostylus. The left and right semispherical gonocoxites fuse mesally and form a distinct median suture in caudo-ventral view (Fig. 5A). They slightly extend upwards dorso-apically, forming a genital concavity. The gonostylus is L-shaped with the distal half abruptly slender and extends dorsally against the lateral side of the aedeagal complex. A protuberance is formed at the bending position fitting into the lateral concavity of the phallobase (Fig. 5A). The stylocavernula *sensu* Tjeder [46], an oval concave area on the caudal side of the basal gonostylus, is furnished with dense sensilla chaetica (Fig. 5B).

#### The Aedeagal Complex

The aedeagal complex protrudes from the genital capsule postero-dorsally (Fig. 5A), and is composed of an epiphallus, a phallobase, a pair of aedeagal lobes, and an elongated penisfilum (Fig. 5D).

The aedeagal complex has a distinct mesal suture, implying its paired origin (Fig. 5A). The phallobase comprises two lateral lumina and one median lumen (Fig. 5C). The two lateral arms of the epiphallus stretch into these two lateral lumina. The median lumen is a channel to transfer sperm. The sperm channel is broader in diameter at the entrance and gradually becomes narrower afterwards, and finally extends as a penisfilum. The heavily sclerotized penisfilum is a hollow tubular structure, slender and curled like a spring (Fig. 5D).

### Morphology of the Female Genitalia

**Figure 6:**
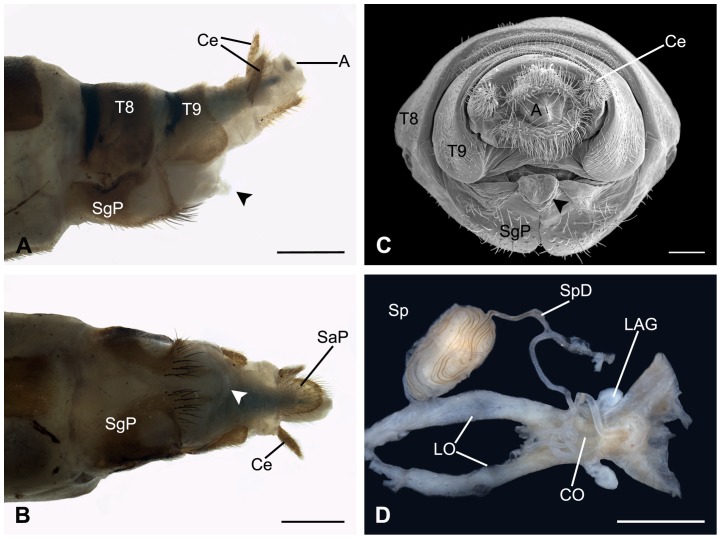
Female genitalia of *B. planus*. (A)–(C) Lateral, ventral, and caudal views of the female terminalia, arrowheads show the membranous structure in the apex of the subgenital plate. (D) Part of the female reproductive system, showing the elongate spermathecal duct. A, anus; Ce, cercus; CO, common oviduct; LAG, lateral accessory gland; LO, lateral oviduct; SaP, subanal plate; SgP, subgenital plate; Sp, spermatheca; SpD, spermathecal duct; T8–9, terga VIII and IX. Scale bars: (A) and (B)  = 0.5 mm; (C)  = 200 μm; (D)  = 0.2 mm.

The external genitalia of the female are degenerated, with only the subgenital plate prominent (Fig. 6A). The subgenital plate is heavily sclerotized and separated mesally by a broad triangular membranous area into two halves (Fig. 6B, C). A glabrous circular- or even oval-like membranous area exists midway on each side of the subgenital plate. The subgenital plate forms the ventral wall of the genital chamber to carry eggs temporarily for insemination prior to oviposition. The thick common oviduct opens into the genital chamber above the terminal part of the subgenital plate (Fig. 6D). A pair of short pouch-like glands (lateral accessory glands) arises from the lateral sides of the common oviduct. The orifice of the spermathecal duct (the copulatory pore) is located above the posterior end of the gonopore (the opening of the common oviduct) on the inner part of the genital chamber. The spermatheca is elliptical in shape, with fine spermathecal duct very long and coiling repeatedly over its surface (Fig. 6D). The spermathecal duct leaves the coils and runs along the dorsal surface of the common oviduct twisty, becoming thicker gradually toward the basal end in diameter (Fig. 6D).

## Discussion

### Co-Evolution of the Face-to-Face Copulation and the Male Genitalia

Male and female hangingflies mate face-to-face in a hanging position. The male genitalia have an extremely elongated penisfilum, paired degenerated gonostyli, a pair of well-developed modified epandrial lobes, and a proctiger. Here, we show that as an intromittent organ through which the ejaculate is transferred to the female spermatheca, the elongated spring-like penisfilum of the male hangingfly is curled dorsally, in a direction opposite to that of the female genitalia when in the face-to-face hanging position, thus it is impossible for the penisfilum to touch the female genitalia unless the male twists his abdomen. Twisting 180° along the male's longitudinal axis of abdomen makes it feasible for the penisfilum to insert into the orifice of spermathecal duct of the female. It is interesting to note that the spermathecal duct and the penisfilum are both very long. During copulation, the penisfilum fully inserts into the reproductive tract of the female (Fig. 3), so that the length of the spermathecal duct of the female must be at least the length of the penisfilum of the male [30], [45]. The elongated penisfilum may have evolved to ensure that male sperm is positioned well within the female reproductive tract, thus increasing male paternity success [17].

Longitudinal twisting of abdomen is an unusual phenomenon in animals [47]. In fact, if an organ is twisted, its function might be blocked or constricted, thus twisting occurs rarely in the animal kingdom except insects [47]. However, twisting of abdomen or genitalia by 180° or even 360° during copulation occurs in many insects, such as Dermaptera, Heteroptera, Hymenoptera, and especially Diptera [47]–[51]. Almost all the males of flies perform these torsions, either through a full 360° or a less drastic 180° twist [47], [50].

A temporary twisting of the male abdomen up to 180° also occurs in the hangingfly *B. italicus* during mating [28]. We suggest that the 180° twist observed in the male abdomen of *B. planus* may have evolved directly from the behavior of delivering a nuptial prey to the female face-to-face during copulation, as in *B. italicus* [28].

To accommodate the extremely elongated penisfilum, the anus-bearing segment (proctiger) is also involved in the evolution of the male genitalia of Bittacidae. The digitate upper and lower branches of proctiger evolved from the supraanal and subanal plates, respectively, assisting the penisfilum (the intromittent organ) to stretch and to enter into the female spermathecal duct during copulation. According to Córdoba-Aguilar [52], who illustrated the sensory-mediated sperm ejection response in a calopterygid damselfly, we suggest that the dense microtrichia in the proctiger probably serve to mediate sperm ejection by frictional movements between the penisfilum and proctiger.

The paired epandrial lobes of the male are modified from the epandrium [53], and are also called clasper-like projections [54], claspers [55], copulobi [44], surgonopods [56], and copulatory lobes [48], indicating its function as claspers to hold the female during copulation. In contrast, the female genitalia are very simple, with only the subgenital plate prominent, relatively smooth, and glabrous. In order to grasp the smooth subgenital plate of the female more easily, the male genitalia have evolved a patch of numerous stout spines along the inner posterior edge of the epandrial lobes to perform the grasping function by increasing friction during the clasping process.

During the evolution of Bittacidae, the male and female adults perform a face-to-face mating position. In such a copulation position, the original gonostyli are hardly in contact with the female genitalia, so that the gonostyli have greatly reduced and their original clasping function has been lost completely [28]. With the twisting of the male abdomen, the clasping function of the original gonostyli has been taken over by the paired epandrial lobes, which are modified from the ninth tergum of the male.

### Possible Reasons for the Evolution of Face-to-Face Mating

Face-to-face mating in a hanging position is unique for hangingflies, but what causes this phenomenon remains unclear. Our data suggest that the face-to-face mating position in hangingflies may arise through sexual conflict and be related to the observed nuptial feeding behavior.

Sexual conflict occurs when the fitness interests of the two sexes are not identical [57]. Males typically try to maximize the number of females mated so as to maximize fertilization success, while females usually develop strategies to decrease or avoid mating costs from potential males [57]. In hangingflies sexual conflict also lies in part over the control over nuptial gifts. It is in the male's interest to reuse the nuptial gift as his fitness is related to the number of females he copulates with. On the other hand, a female benefits directly by consuming the entire nuptial gift. The adopted face-to-face mating position may ensure that the male has the greatest opportunity of retrieving prey leftovers from the female either prior to or after copulation.
